# Valorization of Agricultural Rice Straw as a Sustainable Feedstock for Rigid Polyurethane/Polyisocyanurate Foam Production

**DOI:** 10.1021/acsomega.3c09583

**Published:** 2024-03-08

**Authors:** Roger G. Dingcong, Mary Ann N. Ahalajal, Leanne Christie C. Mendija, Rosal Jane G. Ruda-Bayor, Felrose P. Maravillas, Applegen I. Cavero, Evalyn Joy C. Cea, Kaye Junelle M. Pantaleon, Kassandra Jayza Gift D. Tejas, Edison A. Limbaga, Gerard G. Dumancas, Roberto M. Malaluan, Arnold A. Lubguban

**Affiliations:** † Center for Sustainable Polymers, Mindanao State University − Iligan Institute of Technology, Iligan City 9200, Philippines; ‡ Department of Civil Engineering and Technology, Mindanao State University − Iligan Institute of Technology, Iligan City 9200, Philippines; § Department of Materials Resources Engineering and Technology, Mindanao State University− Iligan Institute of Technology, Iligan City 9200, Philippines; ∥ College of Engineering, Capitol University, Cagayan de Oro City 9000, Philippines; ⊥ AC Joyo Design and Technical Services, Davao City 8000, Philippines; # Department of Chemistry, The University of Scranton, Scranton, Pennsylvania 18510, United States; ¶ Department of Chemical Engineering and Technology, Mindanao State University − Iligan Institute of Technology, Iligan City 9200, Philippines

## Abstract

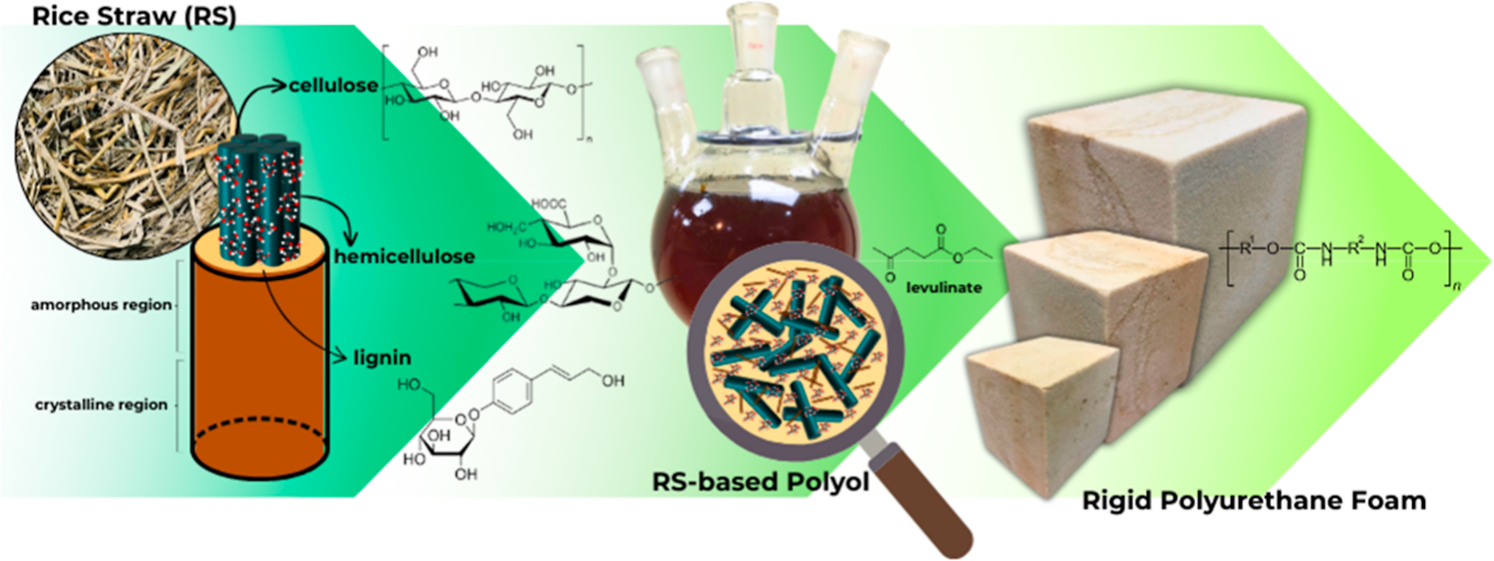

Agricultural rice straw (RS), often discarded as waste in farmlands, represents a vast and underutilized resource. This study explores the valorization of RS as a potential feedstock for rigid polyurethane/polyisocyanurate foam (RPUF) production. The process begins with the liquefaction of RS to create an RS-based polyol, which is then used in a modified foam formulation to prepare RPUFs. The resulting RPUF samples were comprehensively characterized according to their physical, mechanical, and thermal properties. The results demonstrated that up to 50% by weight of petroleum-based polyol can be substituted with RS-based polyol to produce a highly functional RPUF. The obtained foams exhibited a notably low apparent density of 18–24 kg/m^3^, exceptional thermal conductivity ranging from 0.031–0.041 W/m-K, and a high compressive strength exceeding 250 kPa. This study underlines the potential of the undervalued agricultural RS as a green alternative to petroleum-based feedstocks to produce a high-value RPUF. Additionally, the findings contribute to the sustainable utilization of abundant agricultural waste while offering an eco-friendly option for various applications, including construction materials and insulation.

## 1 Introduction

Rice straw (RS), an agricultural byproduct arising abundantly from rice paddy harvests, constitutes a substantial global concern.^1^ As of 2019, the RS annual production is about 100 to 140 t in Southeast Asia, while it is 330 to 470 t across Asia and 370 to 520 t worldwide.^2^ Traditionally, RS residues have been either left to undergo natural decomposition in the fields or incinerated, practices that give rise to pressing environmental and economic issues.^1,3−5^ Natural decomposition in flooded fields produces methane, a potent greenhouse gas, and may impact soil fertility through nutrient tie-up.^6^ While incineration raises environmental alarms with the release of pollutants like particulate matter and greenhouse gases. The subsequent disposal of ash poses challenges to soil and water integrity.^7^ From an economic standpoint, natural decomposition, while seemingly less capital-intensive, carries hidden costs associated with potential yield losses due to pests and diseases, necessitating increased fertilization.^8^ On the other hand, incineration demands significant capital investment for facility establishment, and its viability hinges on energy market dynamics.^9,10^ Consequently, there is an imperative need to explore sustainable methodologies and innovative technologies capable of mitigating environmental footprints while simultaneously augmenting the economic viability of rice production systems.

**Figure 1:**
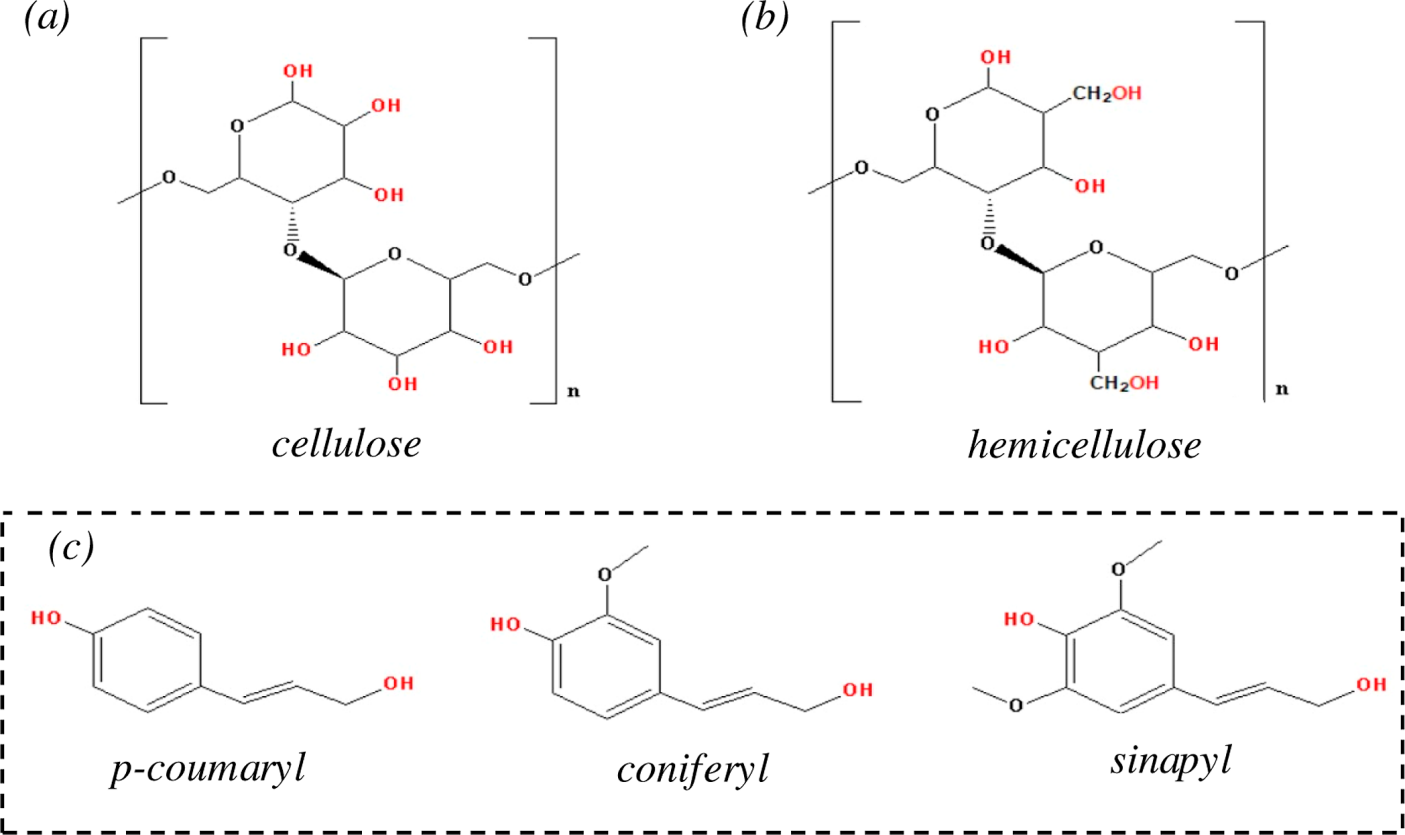
Major components of lignocellulosic biomasses include (a) cellulose, (b) hemicellulose, and (c) lignin.

RS is generally composed of lignocellulosic constituents and encompasses cellulose, hemicellulose, lignin, and other insoluble lignin accounting for roughly 38%, 25%, 12%, and 25% of its composition, respectively.^11−13^ Cellulose, a pivotal structural component, is made of glucose units interconnected by beta-1,4-glycosidic linkages (Figure 1a).^11,14^ In contrast, hemicellulose, a branched polysaccharide, displays a diverse composition of sugar monomers linked by various glycosidic connections (Figure 1b).^15^ While lignin, a complex noncarbohydrate polymer, primarily consists of phenolic compounds linked through carbon-to-carbon and ether bonds (Figure 1c).^16^ The intrinsic hydroxyl groups present in these constituents render RS an attractive resource for polyol production. Polyols obtained from RS liquefaction show great potential as an environmentally responsible alternative to conventional petroleum-based polyols widely utilized in polyurethane (PU) material production.^17−21^ In contrast to alternative sustainable options like vegetable oils, lignocellulosic biomass presents itself as a more economically viable solution owing to its abundance and non-competitive nature with food industries.^22,23^

**Figure 2:**
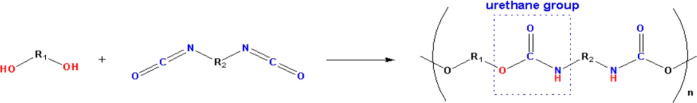
General polyurethane formation mechanism via the reaction of polyol and polyisocyanate.

One of the most well-known PU products is rigid PU foam (RPUF), commonly employed in construction and insulation applications due to its exceptional insulating properties and structural integrity.^24−28^ PU synthesis entails the reaction between hydroxyl (−OH) groups in polyols and –NCO groups in isocyanates,^29,30^ (Figure 2). The polyol types significantly influence the resulting PU properties.^28,31−35^ Traditionally, polyols used in PU production are primarily derived from petroleum feedstocks. However, industrial-scale petroleum-based PU production is associated with substantial adverse environmental impacts, contributing to global environmental hazards.^36,37^ This presents a pressing need to explore alternative and sustainable sources of polyols for PU production considering the environmental and economic challenges associated with petroleum-based polyols.

In recent years, there has been significant attention to the exploration of vegetable oil VO-based polyols as a promising avenue for PU production.^28,38−40^ VOs are generally converted into polyol via functionalization.^41^ VOs with saturated fatty acid chains, such as coconut oil, are usually functionalized via a transesterification/transamidation process,^28^ while VOs with unsaturated fatty acid chains, such as soybean oil, are functionalized via epoxidation followed by oxirane ring opening.^42^ While these polyols demonstrate considerable potential as partial substitutes for petroleum-based counterparts in PU synthesis, the associated high cost of common vegetable oils poses economic challenges for widespread industrial adoption. In parallel, lignocellulosic biomass derived from diverse agricultural wastes has garnered substantial attention as an alternative resource, such as food processing wastes, crop residues, woods, and biorefinery byproducts.^13,43,44^ This biomass is typically converted into polyols either via oxypropylation or liquefaction processes.^44^ The oxypropylation process is usually carried at high pressure (650–1820 kPa) and temperature (100–200 °C) using a base catalyst such as KOH.^44,45^ On the other hand, liquefaction is carried at 150–250 °C at an atmospheric pressure using either acid- or alkali- catalysts.^44,46,47^

Existing literature highlights that RPUFs derived from VOs often exhibit superior thermomechanical properties, attributed to their intrinsic chemical structure, in comparison to those obtained from lignocellulosic biomass.^23^ This distinction is frequently attributed to the inherent difficulty in decomposing lignin, a constituent present in lignocellulosic biomass, during the production process.^19,48^ Despite these observed differences, there are no direct implications for the properties of the resulting RPUFs, as they are contingent upon the specific characteristics of the biomass utilized in each case. The interplay of various factors underscores the nuanced nature of the polyol source materials and their impact on the ultimate properties of rigid polyurethane foams. However, the advantageous utilization of lignocellulosic biomass over vegetable oils lies in its cost-effectiveness, as it is commonly treated as waste in most farmlands.^23^

This study investigates the valorization of underutilized agricultural RS as a substitute for petroleum feedstock in RPUF production, addressing both environmental and economic considerations. The research commenced with the synthesis of polyols derived from RS via a liquefaction process. The RS-derived polyols were utilized as eco-friendly alternatives to traditional petroleum-based polyols for RPUF production. The resulting PU foam samples were subjected to comprehensive characterization, including assessments of their compressive strength, density, and thermal conductivity, to evaluate their suitability for industrial applications in construction and insulation. Additionally, the morphological characteristics of the RPUF samples were examined through scanning electron microscopy (SEM). In addition to assessing the thermo-mechanical properties of RPUF, their thermal behavior was also investigated through thermogravimetric analysis (TGA) and differential scanning calorimetry (DSC).

Notably, the primary advantage of utilizing RS-based RPUF is its abundant availability in rice-farming regions, a readily renewable resource that mitigates waste and reduces environmental burdens. Moreover, this research contributes to green and sustainable PU production by offering an eco-friendly alternative, while concurrently addressing the challenge of underutilized RS residues, both environmentally and economically, bridging the agricultural and industrial sectors toward sustainable and responsible practices.

## 2 Materials and Methods

### 2.1 Materials

The RS biomass was procured from a local rice farm in Lanao del Norte, the Philippines. The polymeric methylene diphenyl diisocyanate (PMDI) (PAPI 27) employed in the study exhibited an NCO content and functionality of 31.4 wt % and 2.7, respectively, and was purchased from Dow Chemicals. Calcium oxide (CaO), silicone surfactants (INV 690 and DABCO DC 5357), zinc oxide (ZnO), foam catalysts (Polycat 8 and Polycat 5), refined glycerol, and petroleum-based polyol VORANOL 490 (V490) (characterized with and hydroxyl (OH) value and functionality of 490 mg KOH/g and 4.3, respectively, and an average molecular weight of 490) was supplied by Chemrez Technologies. The reagent-grade sulfuric acid (H_2_SO_4_) catalyst, deuterated chloroform (CDCl3), and sodium hydroxide (NaOH) employed in the study were procured from Sigma-Aldrich.

### 2.2 Liquefaction of Rice Straw

The RS used in this study was first dried in a drying oven while maintaining a temperature of 110 °C for 48 h and subsequently ground using a knife mill. The ground RS was then sieved through a 50-mesh fraction to obtain a uniform particle size. The liquefaction process was performed in a 250 mL round-bottom 3-necked flask, where a mixture of 12 g of RS, and 120 g of glycerol was introduced while heating and maintaining a constant temperature of 160 °C using a heating mantle. Over 30 min, 2 g of H_2_SO_4_ was gradually introduced to the mixture while continuously stirring at 1500 rpm using a magnetic stirrer to minimize the probability of detrimental recondensation reactions. The temperature was sustained at 160 °C for an additional 3 h as established in the previous studies for an acid-catalyzed liquefaction.^44,49^ Subsequently, the resulting mixture was neutralized to achieve a pH range of 6–7 using sodium hydroxide. After liquefaction, the mixture was then cooled to 50 °C, and the liquefaction residues were separated through filtration. The obtained liquefaction residue was subsequently subjected to a 48 h drying process at 110 °C, and the yield was determined using eq 1.
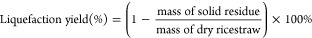
1

### 2.3 Rigid Polyurethane Foam Preparation

The study utilized a modified RPUF foam formulation as previously described by Dingcong et al. (2023),^28^ and the specific components are detailed in Table 1. The PMDI (A-side component) was introduced to the B-side component with an isocyanate index of 110 per part of polyol. The isocyanate index serves as a quantitative representation of the NCO/OH ratio employed in the PU formulation, where an index of 100 signifies a theoretically stoichiometric balance of –NCO to react with –OH groups in the polyol component. Conventionally, a deliberate excess isocyanate index of 10 is strategically utilized in water-blown polyurethane (PU) formulations to serve as a reactant and mitigate the competitive reactivity of residual acids with –OH groups.^50^ This excess isocyanate index ensures the efficient consumption of –NCO groups during polyurethane synthesis, thereby facilitating the desired chemical reactions and promoting the formation of the desired PU structure. The B-side constituents, encompassing the polyols, catalysts, surfactants, and blowing agent, were accurately weighed and mixed in a 500 mL plastic mixing cup. Subsequently, the mixture was subjected to high-speed mixing at 3000 rpm for a duration of 60 s. The resulting blend was then degassed for 120 s. Following this step, polymeric MDI, specifically PAPI 27, was swiftly added to the mixture while continuously stirring for an additional 10 to 15 s at the same rotational speed. Finally, the final blend was then immediately poured into a wooden mold measuring 11.4 × 11.4 × 21.6 cm^3^, lined with aluminum foil, and allowed to expand and solidify under ambient conditions, specifically at 25 °C and 1 atm of pressure. The prepared RS-based RPUFs were labeled according to the percent RS-based polyol replacement to petroleum-based polyol (V490) used in the formulation; RS-0% (0% RS-based polyol, 100% V490), RS-10% (10% RS-based polyol, 90% V490), RS-20% (20% RS-based polyol, 80% V490), RS-30% (30% RS-based polyol, 70% V490), and RS-50% (50% RS-based polyol, 50% V490).

**Table 1:** Formulations for RPUFs at 0%–50% RS-Based Polyol Replacement

		Concentration, php^a^				
Foam Formulation	Components	RS-0%	RS-10%	RS-20%	RS-30%	RS-50%
B-Side Materials						
Polyol	V490	100	90	80	70	50
	Rice-straw Polyol	0	10	20	30	50
Catalyst	Polycat 8	0.5	0.5	0.5	0.5	0.5
	Polycat 5	0.5	0.5	0.5	0.5	0.5
Surfactant	INV 690	1.0	1.0	1.0	1.0	1.0
	DABCO 5357	0.5	0.5	0.5	0.5	0.5
Blowing Agent	Water	2.0	2.0	2.0	2.0	2.0
A-Side Materials						
	Isocyanate index^b^ of PAPI 27	110.0	110.0	110.0	110.0	110.0

a Concentrations of each ingredient are expressed in parts per hundred parts (php) of polyol, adhering to the convention where the cumulative total of all polyols amounts to 100 parts.

b The isocyanate index denotes the ratio of the utilized isocyanate quantity to the theoretically required amount, multiplied by 100.

### 2.4 Analyses of Polyols

The OH value of the produced RS-based polyol was characterized by the ASTM D4274 test method D. The rotational viscosity of the polyol samples was determined using an AMETEK Brookfield DV3T rheometer (Middleborough, MA) following the methods according to ASTM D4878, maintaining torque within the range of 30% to 40%, and maintaining a temperature of 25 ± 0.1 °C. The molecular weight of the polyol samples was determined using an Agilent 1260 Infinity II LC GPC/SEC. The functional group analysis of the polyol was conducted using a Shimadzu IR Tracer 100 spectrometer (Kyoto, Japan). Each sample underwent data collection for 40 scans, spanning wavelengths from 4000 to 400 cm^–^^1^, at a resolution of 2 cm^–^^1^. The ^1^H NMR spectra of the produced RS-based polyol were recorded by using a Bruker Avance 600 MHz cryoprobe NMR spectrometer. Dimethyl sulfoxide (DMSO-*d*_6_) (0.4 mL) was used as a solvent for the RS-based polyol sample (150 mg).^49^

### 2.5 Characterization of Rigid Polyurethane Foam

The RPUF’s thermo-mechanical properties were evaluated at three independent replicates. The thermal conductivity (λ) was assessed using a FOX 200 heat flow meter (Laser-Comp, Wakefield, MA) by ASTM C518, with samples sized at 150 × 150 × 20 mm. The apparent densities of the RPUF specimens were measured by following the guidelines of ASTM D1622. The compressive strengths (σ10%) of the RPUF samples were determined by utilizing a Universal Testing Machine Shimadzu AGS-X Series (Shimadzu Corp., Kyoto, Japan) as per ASTM D1621. Additionally, the characteristic thermal transitions in the RPUF samples were analyzed via DSC using a PerkinElmer DSC 4000 (PerkinElmer, Waltham, MA) at a heating rate of 10 °C/min, employing samples with a weight ranging from 5 to 10 mg. The TGA was conducted using a Shimadzu DTG 60H (Shimadzu Corp., Kyoto, Japan) under a nitrogen (N_2_) atmosphere, at a heating rate of 10 °C/per minute, spanning the range from 45 to 800 °C, with samples weighing between 5 to 10 mg. The cellular structures of the RPUF samples were scrutinized via SEM using a JEOL JSM-IT200 SEM (JEOL, Ltd., Tokyo, Japan). The cell size distribution was analyzed using ImageJ software according to the assessment of each cell’s area.^51^ The average diameter for each cell is determined from the calculated area of each cell, treating each area as that of a perfect circular form.^51−53^

## 3 Results and Discussion

### 3.1 Liquefaction Mechanism

**Figure 3:**
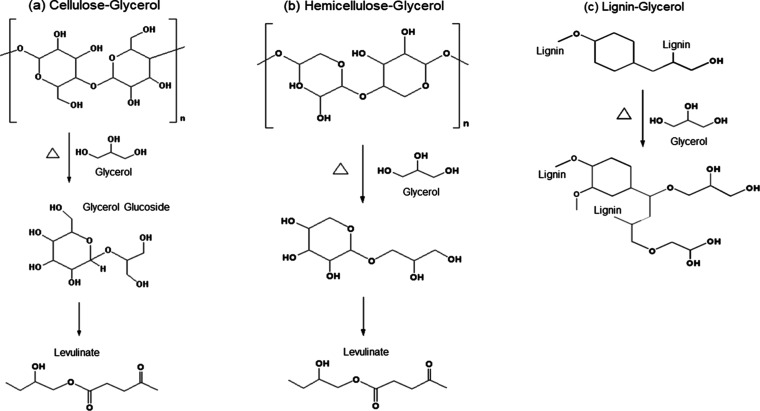
General reaction mechanism for an acid-catalyzed liquefaction of lignocellulosic biomass; (a) cellulose, (b) hemicellulose, and (c) lignin in polyhydric glycerol.

The process of rice straw biomass liquefaction involves the degradation and decomposition of its lignocellulosic components through solvolytic reactions,^44^ as illustrated in Figure 3. The initial stages of liquefaction involve a rapid degradation of hemicellulose, lignin, and amorphous cellulose due to their amorphous structures, facilitating facile interaction with the liquefaction solvents.^44,54^ In contrast, the liquefaction of crystalline cellulose proceeds at a slower rate, extending into later stages, attributed to its densely packed structure, which limits solvent accessibility.^19,55,56^ During solvolysis, cellulose undergoes breakdown into glucose or smaller cellulose derivatives, which subsequently react with the liquefaction solvent, typically glycerol, to yield glycoside derivatives. Subsequently, these glycoside derivatives undergo reactions to form levulinic acid and/or levulinates.^19,55^ It is worth noting that this mechanism follows the established reaction pathways for the liquefactions of other biomass reported in the previous studies.^19,44,55−57^

### 3.2 Characteristics of RS-Based Polyol and Petrochemical Polyol

Table 2 provides a comprehensive comparison between the produced RS-based polyol and the commercial V490, based on critical parameters, including viscosity, OH value, acid number, and liquefaction yield, each analyzed across three independent replicates. Notably, the RS-based polyol exhibits a slightly higher viscosity of 6851 mPa·s compared to V490, which has a relatively lower viscosity of 5590 mPa·s. The relatively elevated OH value of the RS-based polyol (780 mg KOH/g) compared to V490 can be attributed to the substantial presence of free glycerol solvents used in the liquefaction process. While glycerol is considered an eco-friendly alternative to petroleum-based polyols, it is important to recognize the potential trade-offs, including reduced reactivity, adverse effects on mechanical properties, increased foam brittleness, and compatibility challenges with certain blowing agents.^38,40^ Furthermore, the RS-based polyol demonstrates a minimal acid number (3.5 mg of KOH/g), likely associated with residual acid catalysts from the liquefaction process. Lastly, the impressive liquefaction yield of 84.6% underscores the effectiveness of the liquefaction method in converting solid RS biomass into valuable polyol resources for potential use in RPUF production, offering a sustainable and eco-friendly alternative to traditional petroleum-based polyols.

**Table 2:** Properties of RS-Based and Commercial (VORANOL 490) Polyols Employed in RPUF Formulations

	Viscosity (mPa.s) at 25 °C	OH Value (mg KOH/g)	Acid Number (mg KOH/g)	Molecular Weight (Da)	Functionality	Liquefaction Yield (%)
RS-based polyol	6851 ± 82	780 ± 12	3.5 ± 0.4	523	7.3	84.6 ± 3.2
VORANOL 490	5590 ± 75	490 ± 8	0.0 ± 0.0	490	4.3	N/D

**Figure 4:**
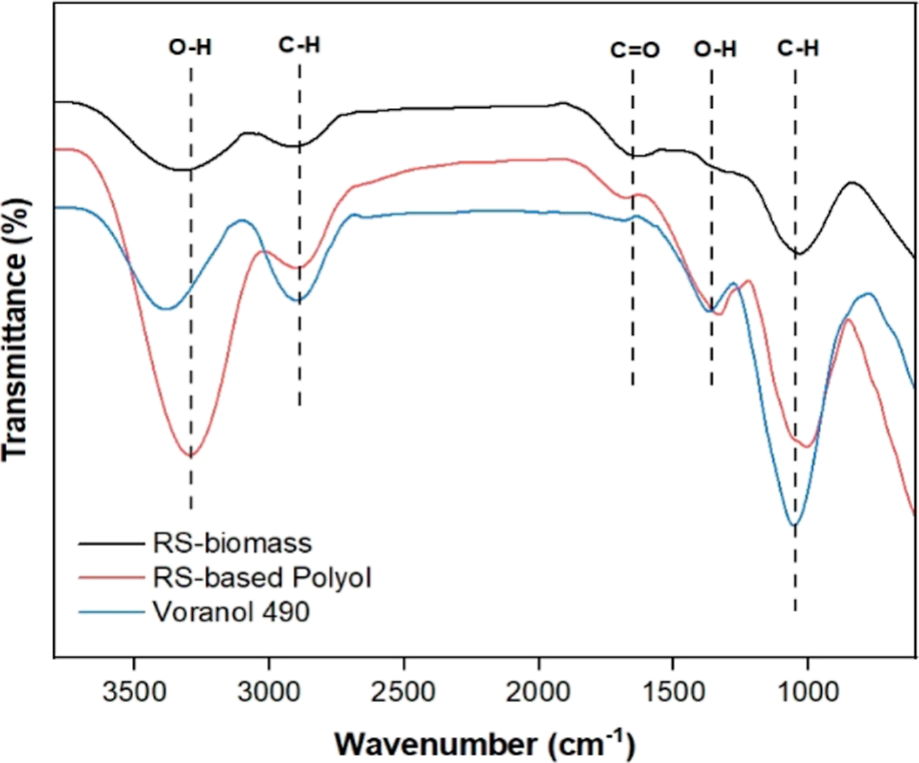
FTIR analysis of RS-biomass, RS-based polyol, and petrochemical polyol (Voranol 490).

The changes in the chemical features of RS biomass during the liquefaction process were evaluated using FTIR analysis by comparing the IR spectra of RS biomass and RS-based polyol as presented in Figure 4. The distinct bands at 3500 to 3300 cm^–^^1^ corresponded to intrinsic hydroxyl groups (−OH) in RS components.^49,58^ The higher intensity of this bond in RS-based polyol compared with RS biomass suggested an improved OH functionality for urethane formation. The IR signal at 1735 cm^–^^1^ represented the C = O bond stretching characteristic of holocellulose present in both samples.^59^ The 2860 cm^–^^1^ band, corresponding to C–H stretching vibrations,^60^ exhibited a notable increase in intensity, indicating the influence of glycerol and a successful occurrence of a chain extension reaction converting RS-biomass into polyol.^49^ The 1710 cm^–^^1^ band associated with C = O groups indicated ether bond dissociation, and the increased band intensity at 1100 cm^–^^1^ confirmed the presence of ether carboxyl (C–OH) and (R-O-R′) groups.^49^ The band from 1240 to 1210 cm^–^^1^ (in RS-based polyol) confirmed hydroxyl (OH) group interaction with liquefaction solvents. Additionally, the FTIR spectrum of the V490 polyol confirms the presence of the petrochemical polyol through the presence of the carbonyl group (C = O) band between 1760 and 1640 cm^–^^1^.^49^ Overall, the FTIR results collectively indicated the successful degradation and dissolution of RS during the liquefaction process, aligning with previous research on agricultural biomass residues.^12,21,49,61^

**Figure 5:**
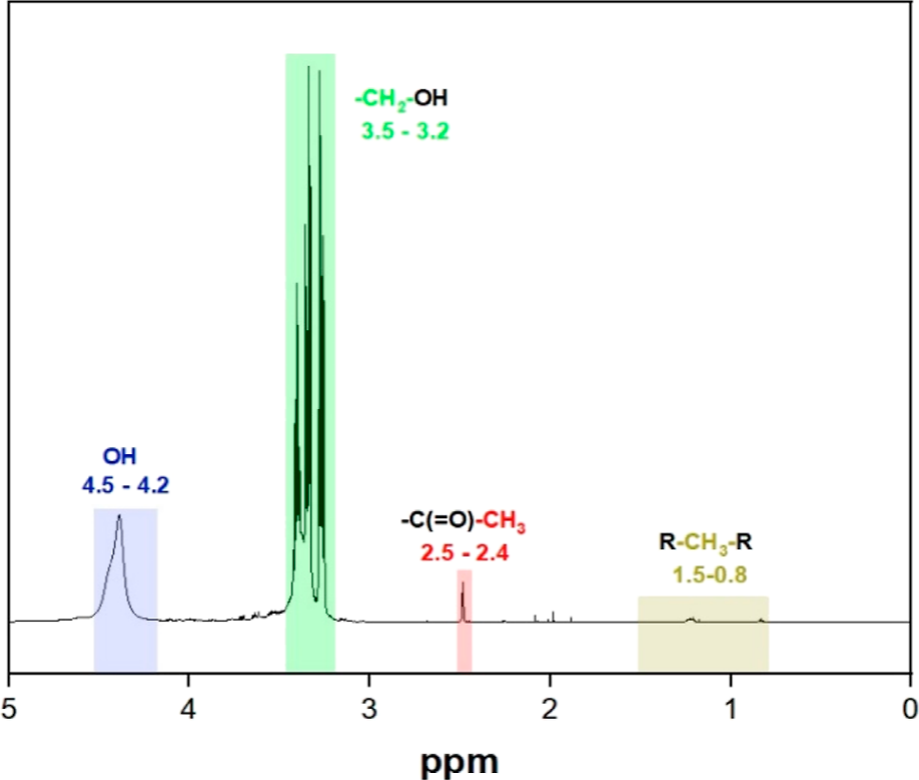
^1^H NMR spectra of RS-based polyol reveal its chemical features.

Figure 5 presents the NMR spectroscopy analysis performed to assess the chemical structural features of the polyol derived from RS. The spectrum reveals distinctive regions, offering insights into the molecular compositions. The aromatic acetyl groups’ protons manifest in the 2.5–2.4 ppm region, and protons located on the aliphatic moiety in lignin components resonate in the 1.5–0.8 ppm region.^49,62^ Moreover, the protons of adjacent methylene groups to the hydroxyl groups originating from both glycerol and RS-based polyol components are found in the 3.5–3.2 ppm region.^63,64^ Finally, the protons associated with the lignin hydroxyl groups are identified in the 4.5–4.2 ppm region, providing conclusive evidence for the successful liquefaction of RS biomass into RS-based polyol.^49^

### 3.3 FTIR Analysis of Rigid Polyurethane Foams

**Figure 6:**
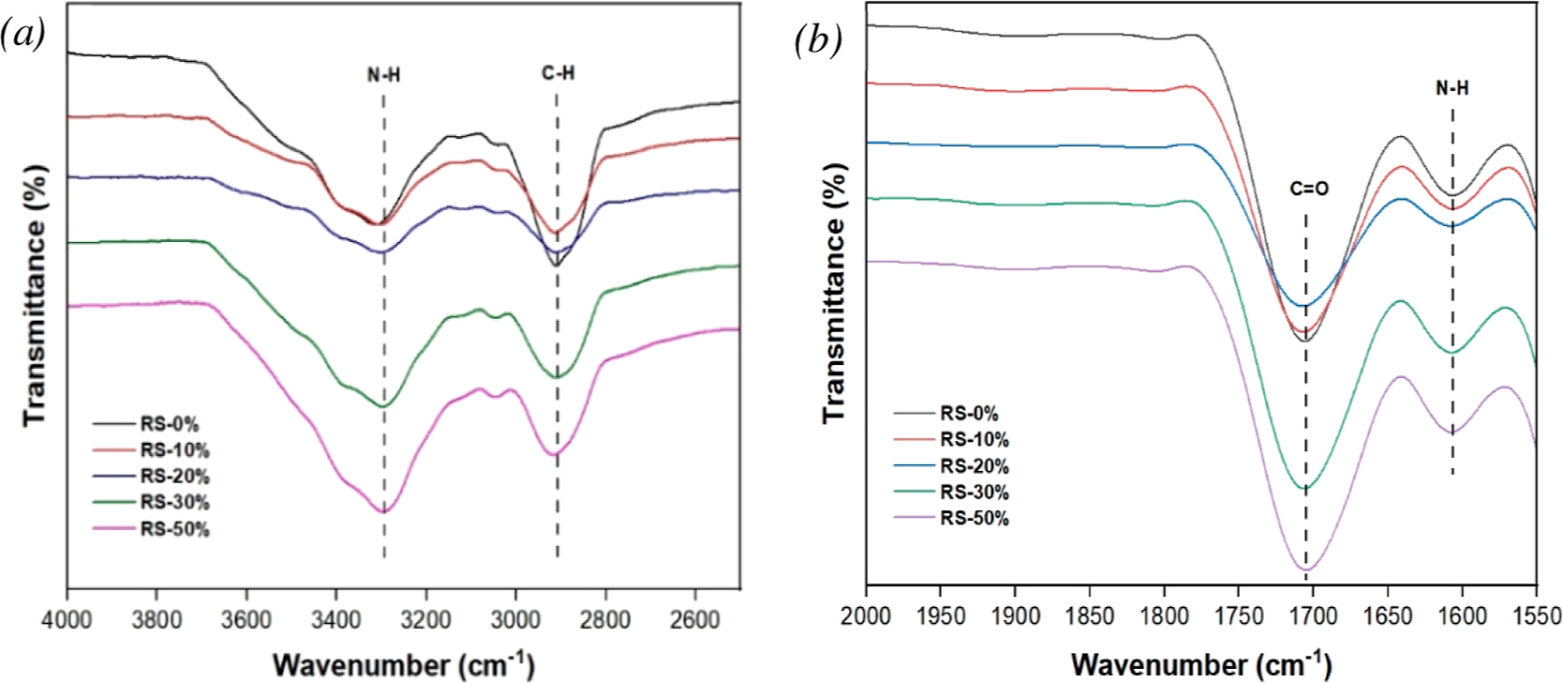
FTIR analysis of the prepared RPUFs at different RS-based polyol weight percent replacement (e.g., 0%, 10%, 20%, 30%, and 50%); (a) FTIR spectra from 4000 to 2500 cm^–1^ and (b) FTIR spectra from 2000 to 1550 cm^–1^.

The FTIR spectrum presented in Figure 6 presents the chemical composition of RPUF samples, particularly focusing on the effects of substituting V490 with an RS-based polyol. Characteristic urethane bonds are identified through bands at 3600–3200 cm^–^^1^ (δ(N–H)) and 3025–2820 cm^–^^1^ (δ(N–H)) in Figure 6a, and 1770–1700 cm^–^^1^ (ν(C = O)) and 1595–1560 cm^–^^1^ (δ(N–H)) in Figure 6b.^19,28,49^ Even with the escalating amounts of RS-based polyol, the fundamental bands maintain their stable positions, indicating minimal changes in the chemical structure of the RPUFs. Notably, the increasing weight ratio of RS-based polyol correlates with heightened band intensity at 3300 cm^–^^1^, attributed to the ν(N = H) of the urethane group, indicative of increased concentrations of formed urethane segments. This correlation is attributed to the higher hydroxyl number of the RS-based polyol (hydroxyl value = 780 mg of KOH/g) compared to the petrochemical polyol (hydroxyl value = 490 mg of KOH/g). Additionally, the decrease in band intensity at 2910 cm^–^^1^ implies a reduction in C–H linkages, revealing a relatively lower concentration of CH bonds in RS-based polyol compared to V490.

### 3.4 Foaming Kinetics of Rigid Polyurethane Foams

The data presented in Table 3 show the impact of substituting V490 with RS-based polyol within the 0–50% range on the foaming reactions kinetics of RPUFs. This analysis highlights key parameters such as cream time, gel time, and maximum temperature (*T*_max_),^30,35^ and has been conducted with three replicates, providing insights into the thermodynamic aspects of the foaming process. Significantly, both cream and gel times exhibit a consistent incremental increase as the proportion of RS-based polyol increases. This trend is attributed to the higher OH number of RS-based polyol (780 mg of KOH/g) in comparison to V490 (490 mg of KOH/g), resulting in an elevated consumption rate of NCO for urethane formation. Consequently, the availability of NCO for the blowing reactions is reduced, leading to a reduction in CO_2_ generation, which is responsible for the 3D cell development during the foaming process.^49^ Another contributing factor to the prolonged cream and gel times is the relatively higher viscosity of RS-based polyol compared to RS-0% (6851 vs 5590 mPa.s). The increased viscosity hinders the expansion of bubble cells, limiting the mobility of CO2 from a solid phase to a gas phase and, in turn, slowing down the foam expansion rate.^19^ As a result of the increased viscosity with higher incorporation of RS-based polyol, both gel and cream times were extended, while the polymerization kinetics is decelerated as evidenced by the reduced maximum temperature (*T*_max_).^49^

**Table 3:** Influence of RS-Based Polyol on Cream Time, Gel Time, and Maximum Temperature (*T*_max_) at Different Percent Replacements from 0% to 50%

	RS-0%	RS-10%	RS-20%	RS-30%	RS-50%
Cream time, s	25 ± 3	26 ± 3	28 ± 3	30 ± 3	33 ± 3
Gel time, s	56 ± 4	59 ± 4	65 ± 5	70 ± 6	82 ± 6
*T*_max_, °C	165 ± 7	158 ± 7	146 ± 6	130 ± 6	115 ± 5

### 3.5 Cellular Morphology of Rigid Polyurethane Foams

**Figure 7:**
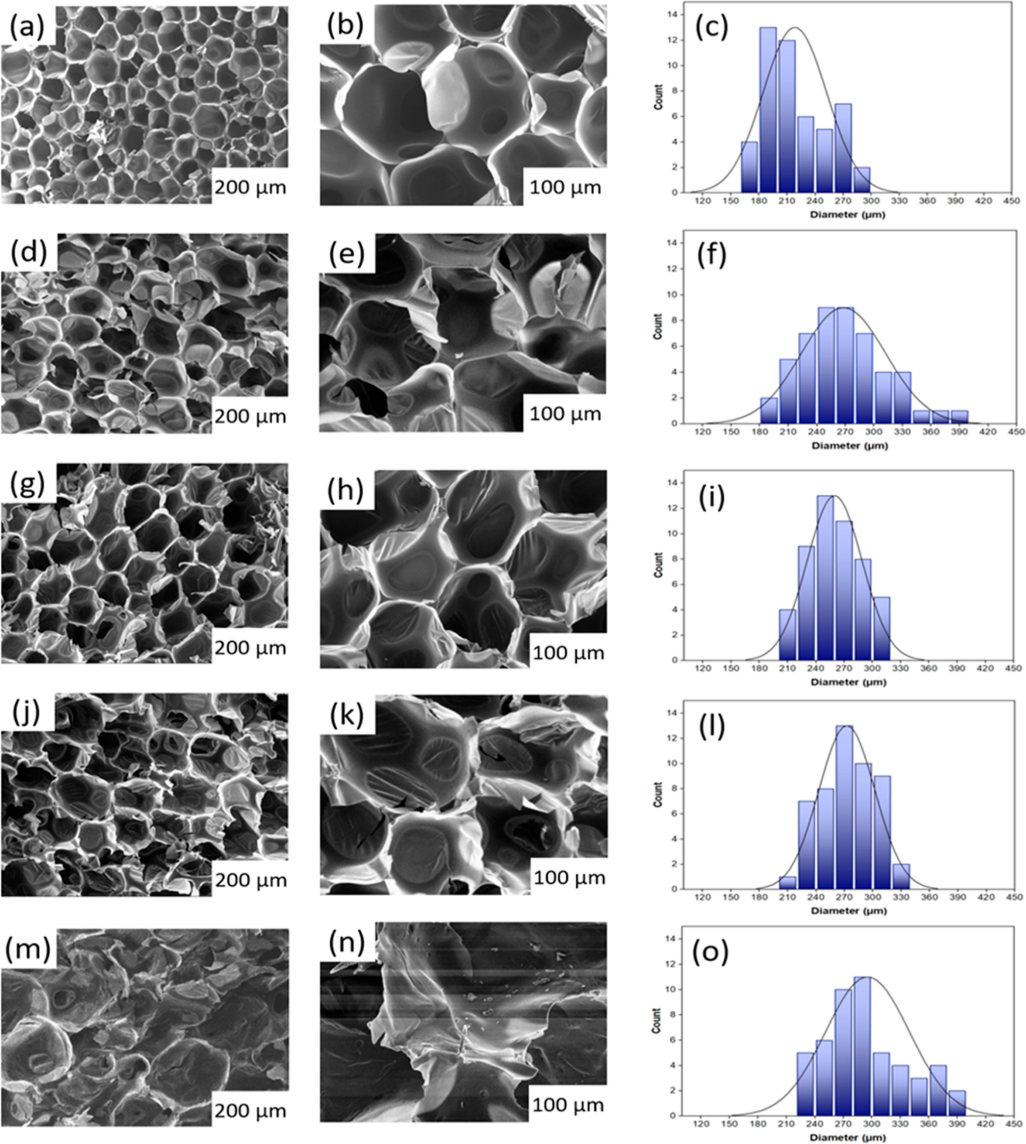
Morphological images and cell size distribution analysis of RPUFs at different RS-based polyol replacements; (a–c) RS-0%, (d–f) RS-10%, (g–i) RS-20%, (j–l) RS-30%, and (m–o) RS-50%.

The investigation of morphological features in RPUFs is pivotal as it directly influences the thermal and mechanical properties.^19,28,31^Figure 7 provides SEM images of RPUFs with varying incorporations of the RS-based polyol. Substantially, as the content of RS-based polyol increases, there is a significant reduction in the regularity of the RPUF’s morphological structures. Furthermore, while the cell size between RS-0% and RS-based RPUFs shows little variation, an increase in cell size is observed in RS-based RPUFs compared to RS-0%, and the distribution of cell sizes becomes less uniform. For instance, RS-0% foams exhibit cell sizes with average diameters of 210 ± 9 μm, while RS-10%, RS-20%, RS-30%, and RS-50% foams feature cell sizes of, 250 ± 7 μm, 265 ± 7 μm, 275 ± 8 μm, and 290 ± 8 μm, respectively. Notably, RPUF containing 50% RS-based polyol displays a broader range of cell size distribution as compared with RS-0%. These changes in the cellular structure can be attributed to the presence of short-chain components in the RS-based polyol such as the glycerol solvent and lignin components. The relatively shorter chain of these components decelerates urethane chain propagation, decreasing the cell wall resistance toward bubble formation thus facilitating the cell expansion.^28,63^ Consequently, an increasing number of cells are ruptured with an increase in the RS-based polyol weight ratio. This can be observed with the significant decrease in the close cell content from 90.12% of RS-00% to 50.04% of RS-50% (Table 4).

**Table 4:** Thermo-Mechanical Properties of the Prepared RPUF Samples at Different Substitutions from 0% (RS-0%) to 50% (RS-50%)

Properties	RS-0%	RS-10%	RS-20%	RS-30%	RS-50%
Compressive Strength, kPa	783 ± 20	575 ± 20	372 ± 18	432 ± 15	255 ± 15
Thermal Conductivity (λ), W/m-K	0.031 ± 0.002	0.033 ± 0.002	0.036 ± 0.003	0.038 ± 0.003	0.041 ± 0.004
Apparent Density, kg/m3	23.5 ± 2.0	22.5 ± 2.0	21.0 ± 2.0	20.3 ± 2.0	18.7 ± 1.5
Closed Cell,%	90.12 ± 5	86.72 ± 4	83.92 ± 4	75.71 ± 3	50.04 ± 3

### 3.6 Mechanical Properties of Rigid Polyurethane Foams

The petrochemical polyol replacement with RS-based polyol emerges to be a crucial factor influencing the mechanical properties of RPUFs as presented in Table 4. Notably, an increase in the proportion of RS-based polyol from 0% to 50% results in a significant reduction in compressive strength (σ10%), with values decreasing from 782.78 to 255.43 kPa. This mechanical deterioration is attributed to distinct morphological changes. First, the augmented presence of open cells in RPUFs with higher RS-based polyol weight ratios contributes to reduced mechanical strength.^28,63^

Second, the increasing average cell sizes of RPUFs with higher RS-based polyol weight ratios lead to lower apparent density, consequently compromising the foam’s compressive strength.^65^

While the mechanical properties of RS-based RPUFs are slightly inferior to those of RS-0%, it is worth noting the great potential they offer as a partial replacement for cleaner and more sustainable RPUF production. The key distinction lies in the renewable and sustainable nature of RS feedstocks, which stand in contrast to the finite resources associated with petroleum feedstocks and their environmental challenges.

### 3.7 Thermal Conductivity of Rigid Polyurethane Foams

The λ plays an essential role in determining the suitability of RPUFs for thermal insulation applications.^66−68^ In the context of rigid foam insulation, lower thermal conductivity values are desirable since they indicate that the material is less effective at conducting heat.^27^ For instance, polyurethane and thermoplastic-based rigid foam are preferred over concrete-based materials as wall insulation due to their relatively low thermal conductivity (0.02–0.1 vs 0.08–2.5 Wm^–1^K^–1^).^69,70^Table 4 presents the λ values of the RPUFs, with neat RS-0% exhibiting a λ of 0.031 Wm^–1^K^–1^. However, with an increasing weight ratio of RS-based polyols, the λ values exhibit an upward trend. For RS-50%, the λ increases from 0.031 to 0.041 Wm^–1^K^–1^, representing a 32% rise compared to neat RS-0%. It is well-established in prior research that the λ of RPUFs is closely tied to their cellular morphology, including factors such as cell anisotropy, size, and shape.^71−73^ The introduction of RS-based polyol disrupts the uniformity of cell structures, resulting in a decrease in the number of closed cells, which in turn increases radiative heat transfer (λr), as observed in the SEM images (Figure 7). Furthermore, it is worth noting that CO^2^ has a lower thermal conductivity (0.015 Wm^–1^K^–1^) than air (0.025 Wm^–1^K^–1^), which signifies that an increase in the number of open cells can result in higher λ values. Additionally, the presence of residual RS particles within the RPUF structure leads to increased λs, explaining the relatively high thermal conductivity of RS-based RPUFs. Furthermore, the structural characteristics of RS-based RPUFs with higher open cell content are potentially due to the partial collapse of the hyperbranched polymer within the pores. The decrease in the quantity of closed cells filled with CO_2_ affects heat transfer within the solid phase.^49^ This result can be attributed to the existence of lignin in the RS composition as well as the more branched structure of RS-based foams, which enhances their thermal stability.

### 3.8 Thermal Behavior of Rigid Polyurethane Foams

**Figure 8:**
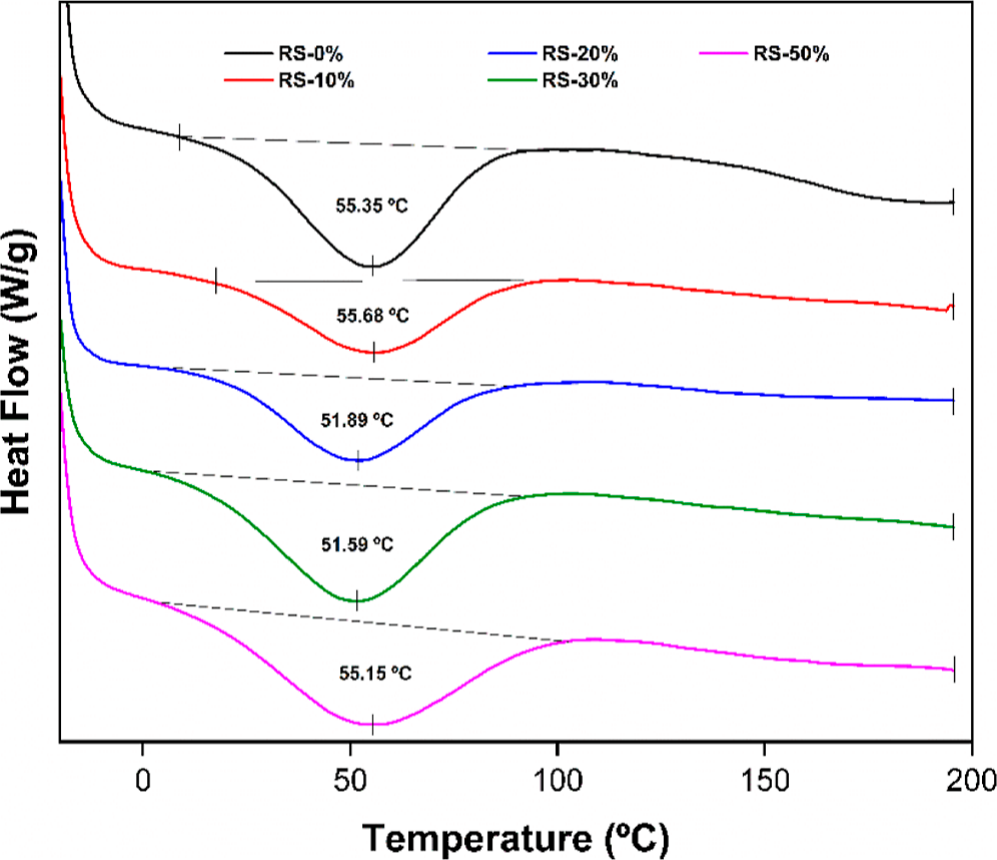
DSC plot of RPUF’s glass transition temperature (Tg) corresponding to the effect of different levels of RS-based polyol replacement.

The thermal transition temperature of the prepared RPUF samples was examined in the DSC analysis, as shown in Figure 8. A substantial thermal transition of RS-0% thermograms can be observed in the range of 14 °C – 90 °C. These transition temperatures indicate the hydrogen bond that takes place at the glass transition temperature (*T*_g_) of the urethane segments.^28,74^ Additionally, all RS-based RPUF foam samples exhibit comparable thermal transitions, as well. The negligible alterations observed in this transition temperature behavior indicate that the introduction of RS-based polyol does not compromise the thermal stability of the RPUF.

**Figure 9:**
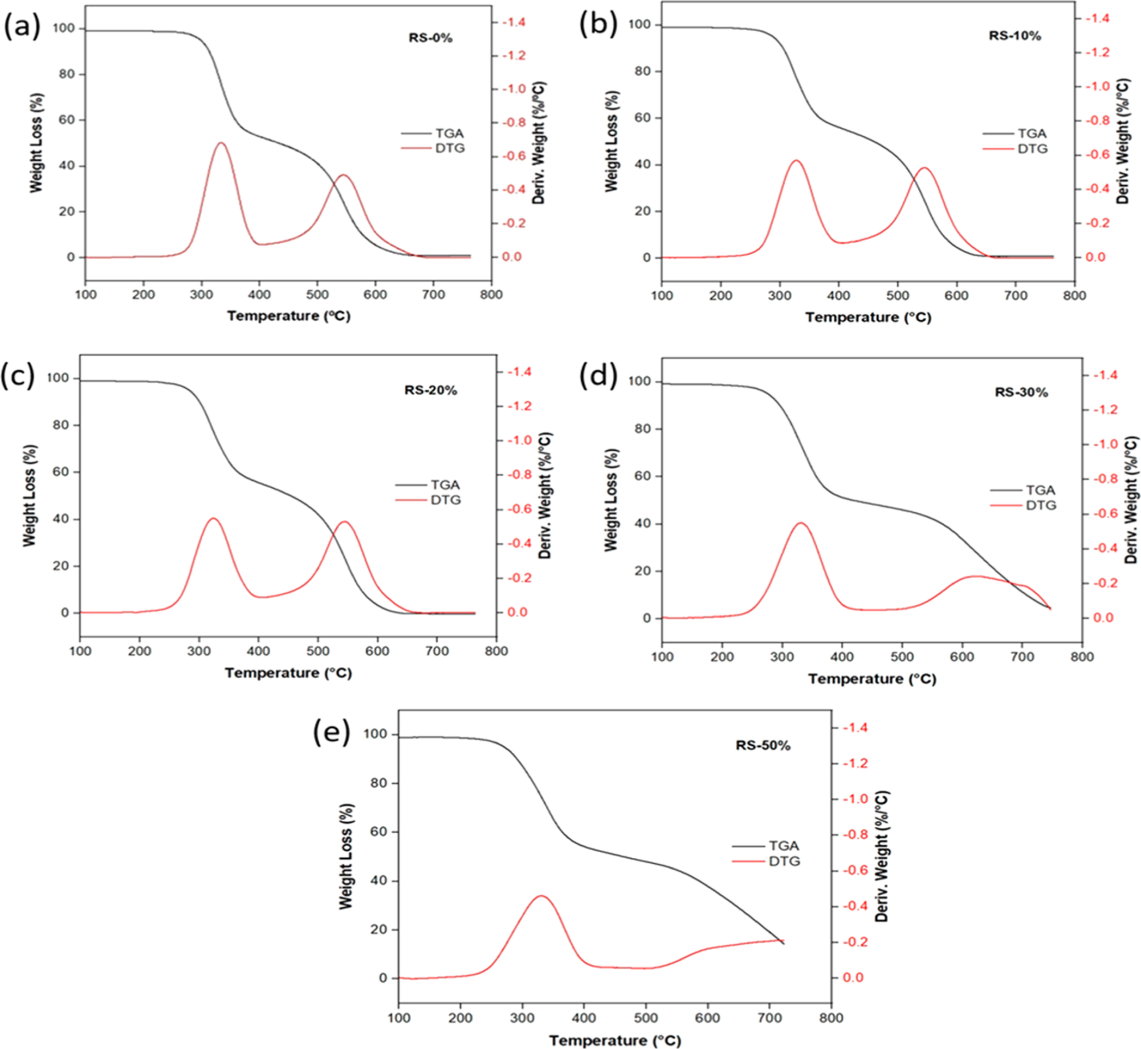
TGA/DTG curves RPUFs at different RS-based polyol replacement; (a) RS-0%, (b) RS-10%, (c) RS-20%, (d) RS-30%, and (e) RS-50%.

Typically, the thermal decomposition process of conventional RPUFs can be classified into three distinct stages. The initial decomposition, between 150 and 350 °C, results in approximately 10% weight loss and corresponds to the breakdown of the hard segment urethane bonds.^75,76^ Additionally, decomposition takes place between 330 and 400 °C, leading to approximately 50% weight loss, primarily attributed to the thermal degradation of the soft segments present in the polyol.^76^ The degradation of the polyol fragments during the soft segment degradation takes place at 500–600 °C, resulting in an 80% weight loss.^75,77^ As depicted in the DTG curves in Figure 9, the RPUF samples collectively demonstrate a thermal degradation temperature range spanning from 260 to 400 °C, with a degradation peak temperature of around 300 °C, indicative of favorable thermal stability. This peak corresponds to both the first and second decomposition stages of the hard and soft segments of RPUF samples. In the third degradation stage, it pertains to the thermal degradation of RS-based components—cellulose, hemicellulose, and lignin.^17^ As the weight ratio of RS-based polyol rises, a proportional increase in mass loss occurs, which can be attributed to the elevated concentration of RS-based polyol and the enhanced miscibility of aromatic soft and hard segments.^31,78^ Moreover, with increasing weight % replacement of RS-based polyol, a gradual mass loss is observed in the second degradation stage starting at around 500 °C. This observation is pronounced in RS-30% and RS-50%. According to Hu et al. (2014),^47^ the rigid structure of lignin and the formation of cross-linked networks during the preparation of RPUF contribute to an enhancement in the heat resistance of the foams with increased incorporation of RS-based polyol. Comparable results have been reported in previous studies as well.^79,80^

## 4 Conclusions

In conclusion, the present study aimed to explore the valorization of underutilized agricultural RS as an economical and sustainable alternative to petroleum-based feedstocks for the production of RPUFs. The synthesis of RPUF samples involved the liquefaction of RS biomass and the subsequent use of the resulting polyol as a partial replacement for petroleum-based polyol in the foaming process. FTIR and NMR analyses confirmed the successful liquefaction of RS biomass, yielding a functional RS-based polyol. The systematic substitution of petrochemical polyol with RS-based polyol in varying proportions resulted in RPUF with apparent densities ranging from 18 to 24 kg/m^3^. These foams exhibited compressive strengths between 255 and 575 kPa, coupled with remarkably low thermal conductivity values of 0.031–0.041 W/(m K). Importantly, thermal stability was not compromised. The study demonstrates that up to 50% of petroleum-based polyol can be effectively replaced with RS-based polyol in the production of RPUFs for thermal insulation applications. Thus far, RS-based RPUFs in this study exhibit the highest capacity to replace petroleum-based polyols during RPUF production compared to other lignocellulosic-based RPUFs. Coupled with its abundance, this implies a great potential for the utilization of RS biomass in industrial-scale RPUF production. This research contributes to the advancement of eco-friendly practices in materials science and encourages the utilization of agricultural residues in the pursuit of a greener and economically viable RPUF production.
